# Prognostic significance of preoperative lymphocytes, albumin, and neutrophils (LANR) index in resectable pancreatic ductal adenocarcinoma

**DOI:** 10.1186/s12885-024-12329-z

**Published:** 2024-05-07

**Authors:** Jiaru Zhuang, Shan Wang, Yuan Wang, Yibo Wu, Renjing Hu

**Affiliations:** 1 Department of Laboratory Medicine, Jiangnan University Medical Center (Wuxi No People’s Hospital), 68 Zhongshan Road, Wuxi, Jiangsu 214000 China; 2 Human reproductive medicine center, Affiliated Hospital of Jiangnan University, 1000 Hefeng Road, Wuxi, Jiangsu 214026 China

**Keywords:** Pancreatic ductal adenocarcinoma, LANR, Biomarker, Prognosis, Survival

## Abstract

**Purpose:**

The index composed of preoperative lymphocytes, albumin, and neutrophils (LANR), a new composite score based on inflammatory response and nutritional status, has been reported to be associated with the prognosis of multiple types of cancer, but the role of LANR in the prognosis of resectable pancreatic ductal adenocarcinoma (PDAC) has not yet been elucidated.

**Patients and methods:**

The data of 142 patients with PDAC who underwent radical resection in the Affiliated Hospital of Jiangnan University from January 2015 to December 2018 were retrospectively analyzed. Receiver Operating Characteristic (ROC) curves were generated to determine the optimal cut-off values for these parameters, as well as the sensitivity and specificity of LANR in predicting survival. The Kaplan–Meier method was used to draw the survival curves. Log rank test was used for univariate analysis, and Cox proportional hazards regression model was used for multivariate analysis.

**Results:**

The optimal cut-off value of LANR was 18.145, and a low preoperative LANR was significantly correlated with the location of the tumor (*p* = 0.047). Multivariate analysis showed that tumor differentiation degree (HR:2.357, 95%CI:1.388–4.003,*p* = 0.002), lymph node metastasis (HR:1.755, 95%CI: 1.115–2.763, *p* = 0.015), TNM stage (HR:4.686, 95%CI: 2.958–7.425, *p* < 0.001), preoperative cancer antigen 19 − 9 levels (HR:1.001, 95%CI: 1.000-1.001, *p* < 0.001) and preoperative LANR (HR:0.221, 95%CI: 0.111–0.441, *p* < 0.001) were independent risk factors for a poor prognosis in patients undergoing radical resection of PDAC.

**Conclusion:**

This study found that preoperative LANR can be used to assess the prognosis of radical resection in patients with PDAC; those with low preoperative LANR had a worse outcome.

## Introduction

Pancreatic ductal adenocarcinoma (PDAC) is the most common primary cancer of the pancreas and its incidence is steadily increasing with 10 to 15 new cases per 100,000 person-years in Western countries [1]. With pancreatic cancer-related deaths increasing year on year, it is estimated that it will become the second leading cause of cancer-related deaths in the United States by 2030 [2]. Data from the National Cancer Institute Surveillance and Epidemiology Database indicate that the five-year overall survival rate for pancreatic cancer patients in the United States is a dismal 10% [3]. As of now, radical surgical resection is still a significant method, or even the only one, to significantly prolong the survival period of pancreatic cancer patients [4]. Although the concept of integrated treatment based on surgery is constantly practiced and updated in clinical work, the 5-year overall survival (OS) rate in patients with radical resection is still less than 20% [5]. In addition, only 20% of pancreatic cancer patients can be treated with surgery [6]. The poor prognosis of pancreatic cancer patients is related to the early asymptomatic nature of the disease, which leads to a late diagnosis and a high possibility of distant metastasis of pancreatic cancer in the early stage [7].

Owing to the grim outlook of pancreatic cancer and the possibility of patients declining treatment, it is imperative to explore simple and inexpensive biomarkers to assist in forecasting patient overall survival, particularly for those who can have surgery [8]. If a patient with a poor prognosis can be identified preoperatively, individualized preoperative therapy such as neoadjuvant therapy may be an option to improve prognosis. However, there are limitations in predicting the prognosis after radical resection in patients with pancreatic cancer by conventional TNM staging alone [9]. Therefore, more biomarkers are needed to help guide treatment and improve the accuracy of prognostic assays for individual patients.

Recent research has demonstrated the impact of systemic inflammatory factors and nutritional status on cancer prognosis [10]. Examples of such indicators include lymphocytes [11], monocytes [12], neutrophils [13], C-reactive protein (CRP) [14], and albumin levels (ALB) [15], To further improve the accuracy of cancer prognosis prediction, a combination of these biochemical indicators, such as the modified Glasgow Prognostic Score (mGPS) [16],C-reactive protein to albumin ratio [17],and neutrophil-to-lymphocyte ratio (NLR) [18], have been studied. A recent study analyzed the research hotspots in the field of immunonutrition and oncology over the past 25 years, exploring the potential of immunonutrition in cancer prevention, treatment, and improved outcomes [19]. Based on this, it is necessary to further study the potential of immunonutrition to affect cancer prognosis. Furthermore, evidence has shown that the new LANR index is a reliable predictor of overall survival following radical surgery in patients with colorectal cancer [20] and stage IA-IIB cervical cancer [21]. Nevertheless, the prognostic significance of combining lymphocytes, albumin, and neutrophils (LANR) in patients with pancreatic ductal adenocarcinoma (PDAC) undergoing radical resection has yet to be thoroughly examined. This study therefore retrospectively analyzed the preoperative blood biochemical indexes of 142 PDAC patients to explore the value of LANR on the survival and prognosis of PDAC patients.

## Materials and methods

### Study design and population

We retrospectively collected data on patients with pathologic diagnosis of pancreatic ductal adenocarcinoma who underwent radical resection at the Affiliated Hospital of Jiangnan University from January 2015 to December 2018. The inclusion criteria were: [1] Pathologic diagnosis of pancreatic ductal adenocarcinoma; [2] no distant metastases confirmed by computed tomography (CT) or magnetic resonance imaging (MRI); [3] first-time radical resection; [4] complete clinicopathological and laboratory data; [5] complete follow-up data. The exclusion criteria were: [1] a history of malignancy or other primary tumors; [2] preoperative neoadjuvant therapy; [3] the presence of acute or chronic inflammatory diseases (such as chronic renal failure, chronic inflammatory bowel disease, diabetes, etc.); [4] anemia or other hematologic diseases. Finally, our study included 142 patients with pancreatic ductal adenocarcinoma who underwent radical resection for statistical analysis.

### Data collection

The clinicopathological information and preoperative blood biochemical indexes of PDAC patients in our hospital were collected through electronic medical records. These included gender, age, BMI, tumor size, degree of differentiation, TNM stage, lymph node metastasis, peripheral nerve invasion, vascular cancer thrombus, tumor location, CEA, CA199, CA125, neutrophils, lymphocytes, and albumin. TNM staging was defined according to the American Joint Committee on Cancer TNM Classification Criteria, Eighth Edition [22].LANR, calculated as lymphocyte × albumin / neutrophil, was also included in the data. Blood samples were collected from all patients at the time of admission.

### Ethics statement

This study was conducted in accordance with the ethical standards of the Declaration of Helsinki of the World Medical Association (9th edition in July 2018) and was approved by the Medical Ethics Committee of the Affiliated Hospital of Jiangnan University (Grant No: JNMS022023058). Since all data is anonymized and aggregated, the Ethics Committee waived the requirement of informed consent.

### Treatments and follow-up

All patients underwent radical surgery for PDAC at the Affiliated Hospital of Jiangnan University, including pancreatic resection and distal pancreatectomy combined with splenectomy. All patients were followed up by reviewing outpatient medical records and telephone follow-up. Patients were followed up every 3 months for the first two years and every 6 months thereafter, and the results of physical examination, laboratory tests, and imaging examinations were recorded in detail. The date the patient underwent surgery was the beginning of the follow-up period, and the OS time was defined as the time from the date of surgery to the date of death or the date of the last follow-up visit.

### Statistical analysis

Continuous data with a normal distribution were presented as mean ± standard deviation (Kolmogorov-Smirnov test, *p* > 0.05). A Chi-square test and independent samples t-test were used to compare clinicopathological features at baseline. The Kaplan-Meier method was used to calculate the cumulative survival rate, and the log-rank test was used to compare survival curves. The multivariate Cox proportional hazards model was used to further test prognostic factors that proved to be statistically significant in univariate analyses, and the results were calculated with 95% confidence interval (CI), with a two-tailed *p* < 0.05 considered statistically significant.

## Results

### Patient characteristics

A total of 142 patients with PDAC who underwent radical resection in the Affiliated Hospital of Jiangnan University from January 2015 to December 2018 were included in this retrospective study, with a median follow-up time of 17.5 months. A total of 123 patients died during follow-up, with an estimated median OS of 21.4 months (range: 2–59 months). We analysed the clinical parameters of all patients who met the inclusion criteria and found that the median age at diagnosis was 64 years (range: 32 to 87 years), of which 62 (43.7%) were over 64 years of age. Of these patients, 84 (59.2%) were male, and 88 (62%) had tumors located in the head or neck of the pancreas. Thirty-one patients (21.8%) had peripheral nerve invasion, 34 (23.9%) had vascular cancer thrombi, and a total of 126 patients (88.7%) had a histologically diagnosed moderately differentiated tumor. Only 33 patients (23.2%) were classified as stage III according to TNM staging criteria. Details of the patients’ baseline characteristics are shown in Table 1.

**Table 1:** Analysis of the clinicopathological characteristics of 142 patients with pancreatic ductal adenocarcinoma

Variables	Total	LANR ≥ 18.145	LANR < 18.145	*P*-value
Patients	142(100)	50(35.2)	92(64.8)	
**Sex**				0.714
Male	84(59.2)	32(64.0)	56(60.9)	
Female	58(40.8)	18(36.0)	36(39.1)	
**Age (years)**				0.323
≤ 64	80(56.3)	32(64)	51(55.4)	
>64	62(43.7)	18(36)	41(44.6)	
BMI (kg/m^2^)	23.87 ± 3.45	24.34 ± 3.05	23.61 ± 3.64	0.234
**Tumor size, cm**				0.563
≥ 4cm	56(39.4)	15(30)	32(34.8)	
<4cm	86(60.6)	35(70)	60(65.2)	
**Differentiation**				0.37
Poor	16(11.3)	7(14.0)	12(13.0)	
Moderate/well	126(88.7)	43(86.0)	80(87.0)	
**TNM stage**				0.718
I	20(14.1)	8(16.0)	16(17.4)	
II	89(62.7)	24(48.0)	49(53.3)	
III	33(23.2)	18(36.0)	27(29.3)	
**LNM**				0.941
Yes	45(31.7)	16(32.0)	30(32.6)	
No	97(68.3)	34(68.0)	62(67.4)	
**Perineural invasion**				0.468
Yes	31(21.8)	10(20.0)	14(15.2)	
No	111(78.2)	40(80.0)	78(84.8)	
**Vascular cancer embolus**				
Yes	34(23.9)	13(26.0)	22(23.9)	0.783
No	108(76.1)	37(74.0)	70(76.1)	
**CEA**				
≥ 4ng/ml	55(38.7)	25(50)	41(44.6)	0.535
< 4ng/ml	87(61.3)	25(50)	51(55.4)	
**CA199**				0.768
≥ 78.9U/ml	72(50.7)	21(42.0)	41(44.6)	
< 78.9U/ml	70(49.3)	29(58.0)	51(55.4)	
**CA125**				0.379
≥ 16.1U/ml	71(50)	31(62.0)	50(54.3)	
<16.1U/ml	71(50)	19(38.0)	42(45.7)	
**Location**				**0.047**
Head	88(62.0)	41(82.0)	61(66.3)	
Body and tail	54(38.0)	9(18.0)	31(33.7)	
Neu (10^9^/L), Median (IQR)	3.59(2.82–4.69)	2.90(2.31–3.57)	4.16(3.26–5.21)	<0.001
ALB (G/L), Median (IQR)	39.0(35.43–40.80)	40.40(36.85–42.95)	38.45(34.95–40.2)	<0.001
Lym (10^9^/L), Median (IQR)	1.31(1.01–1.70)	1.73(1.30–2.16)	1.15(0.89–1.44)	0.001

Abbreviations: IQR, interquartile range; ALB, albumin; Lym, lymphocyte; Neu, neutrophil; CEA, carcinoembryonic antigen; LNM lymphatic node metastasis. a *p* values of nominal variables and continuous variables were calculated using the χ^2^ test and t-test, respectively. P values < 0.05 are shown in bold.

### Prognostic value of LANR for overall survival

**Fig. 1:**
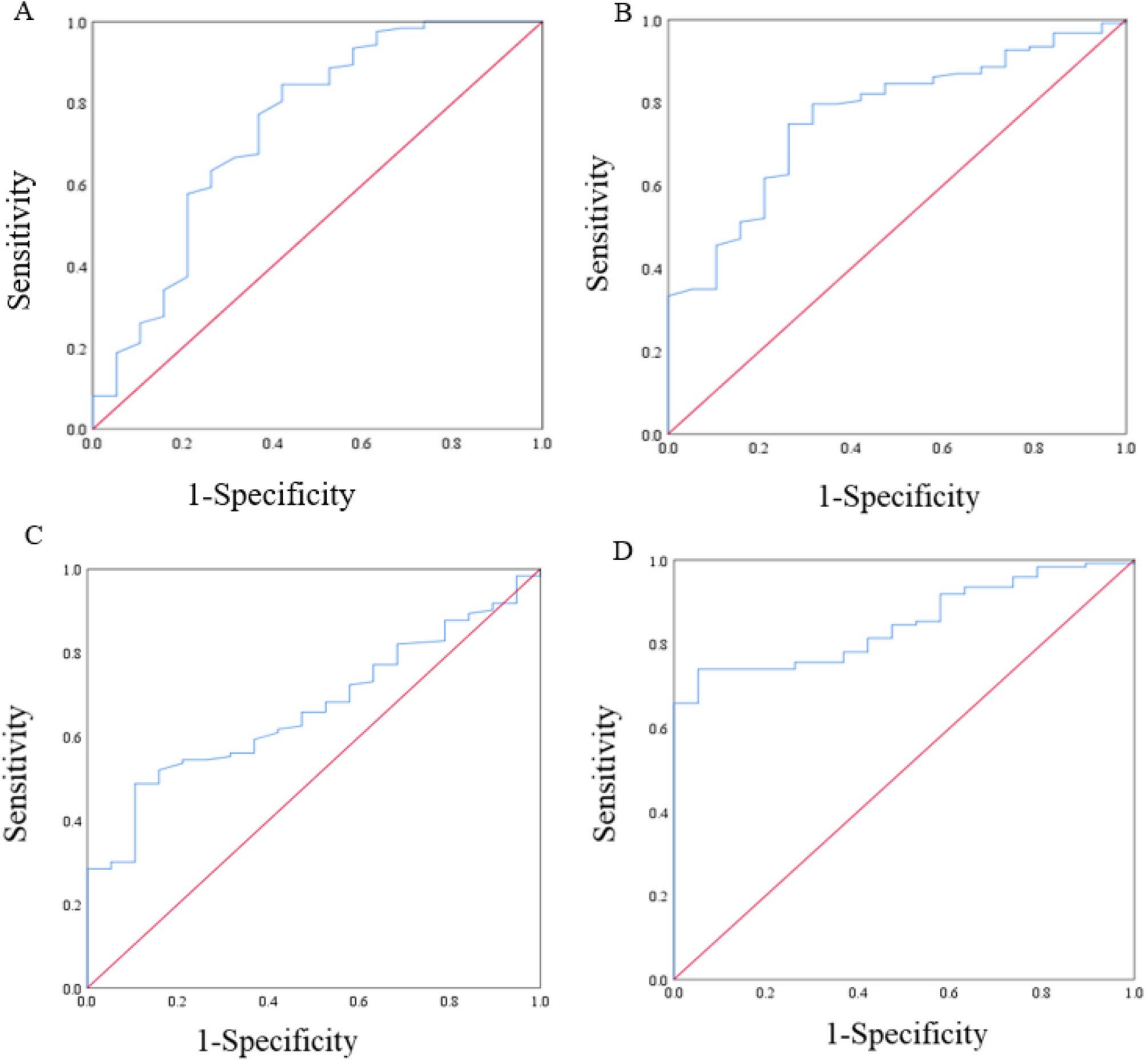
The ROC curve for overall survival of Alb, Lym, Neu, and LANR. **A**: Albumin for OS. **B**: Lymphocyte for OS. **C**: Neutrophils for OS. **D**: LANR for OS.

**Fig. 2:**
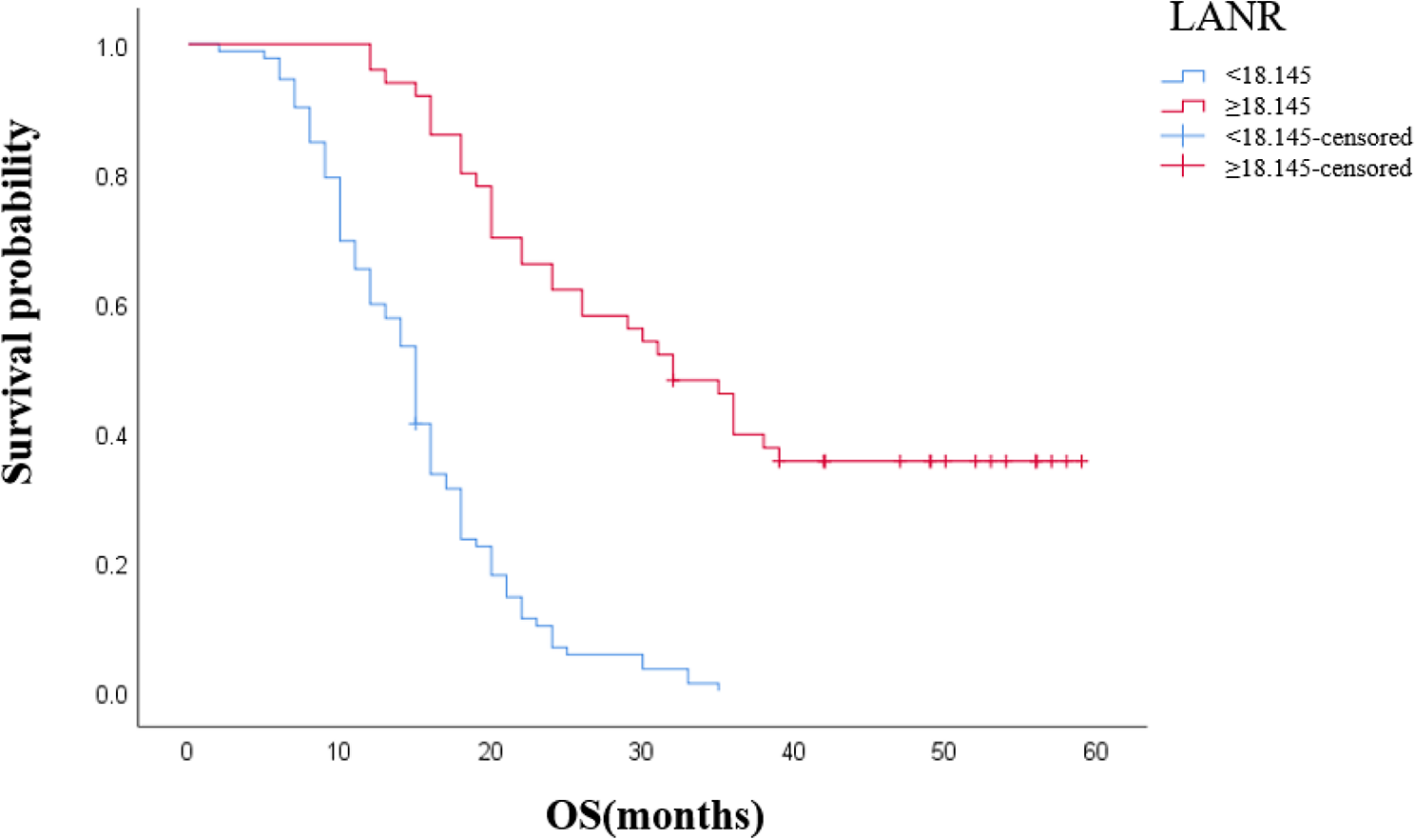
The Kaplan-Meier curves for overall survival of Pancreatic ductal adenocarcinoma based on LANR.

In terms of overall survival, the area under the ROC curve (AUC) and the optimal thresholds for OS for albumin, lymphocytes, neutrophils, and LANR are shown in Fig. 1. Based on the ROC curve, we found that the area under the curve of LANR was the best, with an AUC of 0.816 and an optimal cut-off of 18.145 (sensitivity: 74%, specificity: 94.7%, *p* < 0.001, Table 2). According to the cut-off values, the enrolled patients were divided into high-level (LANR ≥ 18.145, *n* = 50, 35.2%) and low-level (LANR < 18.145, *n* = 92, 64.8%) groups (see Table 1), and the Kaplan-Meier survival curve showed that patients with higher LANR had a longer overall survival (Fig. 2). Univariate analysis showed that TNM stage(*p* < 0.001), degree of differentiation(*p* < 0.001), lymph node metastasis(*p* < 0.001), tumor location(*p* < 0.001), CA199(*p* = 0.01), CA125(*p =* 0.008), albumin(*p* < 0.001), lymphocytes(*p* < 0.001), neutrophils(*p* < 0.001), and LANR(*p* < 0.001) were significantly correlated with OS (*p* < 0.05; Table 2). Multivariate analysis showed that the degree of tumor differentiation (HR:2.357, 95%CI:1.388–4.003, *p* = 0.002), lymph node metastasis (HR:1.755, 95%CI:1.115–2.763, *p*0.015), TNM stage (HR:4.686, 95%CI:2.958–7.425, *p* < 0.001), preoperative cancer antigen 19 − 9 levels (HR:1.001, 95%CI: 1.000-1.001, *p* < 0.001), and preoperative LANR (HR:0.221, 95%CI:0.111–0.441, *p* < 0.001) were independent risk factors for poor prognosis in patients undergoing radical resection with PDAC (Table 2). In patients with resectable pancreatic ductal adenocarcinoma, combined with the area under the ROC curve and multivariate Cox regression analysis, we found that LANR is a reliable predictor of overall survival.

**Table 2:** Univariate and multivariate analyses of overall survival after pancreatic ductal adenocarcinoma.

Variables	AUC	Cut-point	Univariate		Multivariate	
			HR (95%CI)	*P*-value	HR (95%CI)	*P*-value
ALB(G/L)	0.741	41.45	0.886(0.852–0.920)	< 0.001	0.951 (0.902–1.002)	0.058
Lym(10^9^/L)	0.766	1.545	0.350(0.229–0.536)	< 0.001	0.563(0.317–0.999)	0.050
Neu(10^9^/L)	0.666	3.875	1.293(1.182–1.414)	< 0.001	1.116(0.998–1.246)	0.053
LANR	0.816	18.145	0.895(0.870–0.920)	< 0.001	0.221(0.111–0.441)	< 0.001
Sex			1.118(0.934–1.339)	0.224		
Age (years)			1.032(0.723–1.475)	0.861		
BMI (kg/m^2^)			0.966(0.840–1.111)	0.630		
Tumor size, cm			1.162(0.810–1.666)	0.415		
Perineural invasion			0.644(0.374–1.106)	0.111		
TNM stage			5.426(3.830–7.687)	< 0.001	4.686(2.958–7.425)	< 0.001
Vascular cancer embolus			0.992(0.680–1.448)	0.967		
Differentiation			2.792(1.924–4.052)	< 0.001	2.357(1.388–4.003)	0.002
LNM			2.310(1.541–3.463)	< 0.001	1.755(1.115–2.763)	0.015
CEA			1.001(0.993–1.009)	0.847		
CA199			1.001(1.000-1.001)	0.01	1.001(1.000-1.001)	< 0.001
CA125			1.010(1.003–1.017)	0.008		
Location			0.470(0.321–0.689)	< 0.001	2.718(1.533–4.818)	0.071

Abbreviations: HR, Hazard Ratio; CI, Confidence Interval; AUC, Area under the ROC Curve; Lym, lymphocyte; ALB, albumin; Neu, neutrophils; LANR, Lym*Alb/Neu; LNM lymphatic node metastasis

## Discussion

This retrospective study explores the prognostic value of LANR in patients with resectable pancreatic ductal adenocarcinoma and evaluates the relationship between LANR and other clinicopathological factors. The results of the analysis showed that low preoperative LANR was associated with poor prognosis of patients with resectable pancreatic cancer, and the median survival time of patients with LANR < 18.145 was significantly shorter than that of patients with LANR ≥ 18.145. Univariate and multivariate analyses showed that LANR ≤ 18.145 was an independent risk factor for poor prognosis in patients with resectable pancreatic cancer. LANR was significantly correlated with tumor location, and the proportion of pancreatic head tumors in the low LANR group was significantly higher than that in the high LANR group. Pancreatic head cancer can cause obstructive jaundice and has a more severe local inflammatory response, which may affect the nutritional status of the patient [23].Inflammation and malnutrition increase the risk of postoperative adverse events such as CRPOPF [24].

In this study, we investigated the predictive value of 12 clinicopathologic variables (gender, age, tumor size, degree of differentiation, tumor location, TNM stage, lymph node metastasis, vascular cancer thrombus, peripheral nerve invasion, CEA, CA199, CA125) for resectable pancreatic ductal adenocarcinoma, and many attempts have been made to determine the prognostic factors of PDAC in addition to several well-known factors such as TNM stage and carbohydrate antigen 19 − 9 (CA19-9) [25],In several previous studies, LN status and degree of tumor differentiation were identified as prognostic factors for resectable PDAC [26–28],and these findings are consistent with the results of this study, which suggest that LN status and degree of differentiation are independent prognostic factors in patients with PDAC. In addition, studies have shown that neoadjuvant chemotherapy can inhibit tumor progression and effectively prolong postoperative survival [29].however, there is still a lack of uniform criteria for selecting patients who are candidates for neoadjuvant therapy. In our study, we did not include these patients in order to rule out the effect of preoperative neoadjuvant chemotherapy on our outcomes.

Malnutrition is a common diagnosis among cancer patients and has a considerable effect on the prognosis [29].Progressive malnutrition is strongly associated with the development of cachexia, especially in patients with pancreatic and gastric cancers [30].Epidemiological studies on the relationship between serum albumin levels and survival in cancer patients have found that decreased serum albumin levels are associated with reduced survival [31].The relationship between pretreatment albumin levels and prognosis for head and neck cancer [32],kidney cancer [33], cardia adenocarcinoma [34], and ovarian cancer [35] has been reported. Inflammation is known to play a key role in the initiation, progression, and prognosis of tumors, and lymphocytes can not only effectively recognize tumor cells, but also be stimulated by tumor antigens to transform into sensitized lymphocytes to attack tumor lesions, thereby producing antitumor activity, inhibiting angiogenesis, and improving tumor prognosis [35]. Pancreatic cancer patients are a population with a high nutritional risk and a high prevalence of malnutrition. Current studies and ERAS guidelines primarily support the use of preoperative or perioperative medical nutrition, including immunonutrition, to reduce postoperative infection rates and length of hospital stay in patients with pancreatic ductal adenocarcinoma [36]. We should actively consider the use of preoperative medical nutrition in patients with resectable PDAC to potentially prevent postoperative severe malnutrition and its impact on disease and treatment outcomes. Neutrophils are the most abundant circulating white blood cells in the body and have a pro-inflammatory effect [37].Neutrophils in the tumor microenvironment also have similar functions to myeloid suppressor cells, inhibiting T cell proliferation, binding to tumor cells, and supporting tumor cell cycle progression [38]. In our study, ALB, Lym, and Neu were all associated with OS in PDAC in univariate analysis, but their effect on OS was not statistically significant in multivariate analysis, and although they affected OS, they were not independent prognostic factors. LANR is a conjugated indicator of lymphocytes, albumin, and neutrophils. Compared with these three indicators, the prognostic effect of LANR is more significant.

Therefore, preoperative LANR, as an indicator of the ratio of inflammation level to nutritional status, can be regarded as an independent prognostic factor for patients undergoing radical resection of PDAC. This study has some limitations. first of all, this is a retrospective study with its inherent flaws. Second, due to the relatively small number of patients, we did not divide patients into training and experimental groups for statistical validation, and external validation was lacking, and further research was needed. In our study, we focused on the prognostic significance of preoperative LANR, while the relationship between changes in postoperative LANR and the prognosis of these patients has not been studied. In addition, many patients received multiple treatments for tumor recurrence during follow-up, which also affected OS. Finally, our study included a small number of clinical data, and the results may be more instructive in the future by statistically analyzing patients’ comorbidities and other biochemical data (at least creatinine and liver enzymes) to describe their dynamic changes with disease progression.

## Conclusion

In conclusion, our data suggest that preoperative LANR levels are an independent prognostic factor for resectable PDAC. The LANR index, composed of neutrophils, lymphocytes, and albumin, is a potent serum biomarker and may be a reliable prognostic predictor that can accurately predict the survival of patients with early PDAC resection. Compared with TNM staging alone, the combination of LANR and TNM staging is more accurate in predicting the prognosis of PDAC patients. A multicenter study with a larger sample size is needed to validate the prognostic potential of the LANR index in patients with resectable pancreatic cancer.

## Data Availability

No datasets were generated or analysed during the current study.

## Abbreviations

PDAC: Pancreatic ductal adenocarcinoma.
ROC: Receiver operating characteristic.
AUC: Area under the curve.
ALB: Albumin
Lym: Lymphocyte
Neu: Neutrophil
CEA: Carcinoembryonic antigen
LNM: Lymphatic node metastasis
IQR: Interquartile range
CRP: C-reactive protein
mGPS: Modified Glasgow Prognostic Score
NLR: Neutrophil-to-lymphocyte ratio
OS: Overall survival
MRI: Magnetic resonance imaging
CT: Computed tomography
CRPOPF: Clinically relevant postoperative pancreatic fistula

## References

[1] Latenstein AEJ, van der Geest LGM, Bonsing BA, Groot Koerkamp B, Haj Mohammad N, de Hingh I (2020). Nationwide trends in incidence, treatment and survival of pancreatic ductal adenocarcinoma. Eur J Cancer.

[2] Rahib L, Smith BD, Aizenberg R, Rosenzweig AB, Fleshman JM, Matrisian LM (2014). Projecting cancer incidence and deaths to 2030: the unexpected burden of thyroid, liver, and pancreas cancers in the United States. Cancer Res.

[3] Grossberg AJ, Chu LC, Deig CR, Fishman EK, Hwang WL, Maitra A (2020). Multidisciplinary standards of care and recent progress in pancreatic ductal adenocarcinoma. CA Cancer J Clin.

[4] Kamisawa T, Wood LD, Itoi T, Takaori K (2016). Pancreatic cancer. Lancet.

[5] Kulemann B, Hoeppner J, Wittel U, Glatz T, Keck T, Wellner UF (2015). Perioperative and long-term outcome after standard pancreaticoduodenectomy, additional portal vein and multivisceral resection for pancreatic head cancer. J Gastrointest Surg.

[6] Gillen S, Schuster T, Meyer Zum Buschenfelde C, Friess H, Kleeff J (2010). Preoperative/neoadjuvant therapy in pancreatic cancer: a systematic review and meta-analysis of response and resection percentages. PLoS Med.

[7] Groot VP, Gemenetzis G, Blair AB, Rivero-Soto RJ, Yu J, Javed AA (2019). Defining and Predicting Early recurrence in 957 patients with resected pancreatic ductal adenocarcinoma. Ann Surg.

[8] Conroy T, Pfeiffer P, Vilgrain V, Lamarca A, Seufferlein T, O’Reilly EM et al. Pancreatic cancer: ESMO Clinical Practice Guideline for diagnosis, treatment and follow-up. Ann Oncol. 2023. 10.1016/j.annonc.2023.08.009 37678671

[9] Tong Z, Liu Y, Ma H, Zhang J, Lin B, Bao X (2020). Development, Validation and Comparison of Artificial Neural Network Models and Logistic Regression models Predicting Survival of Unresectable Pancreatic Cancer. Front Bioeng Biotechnol.

[10] Caputo D, Quagliarini E, Coppola A, La Vaccara V, Marmiroli B, Sartori B (2023). Inflammatory biomarkers and nanotechnology: new insights in pancreatic cancer early detection. Int J Surg.

[11] Zhou T, Man Q, Li X, Xie Y, Hou X, Wang H (2023). Artificial intelligence-based comprehensive analysis of immune-stemness-tumor budding profile to predict survival of patients with pancreatic adenocarcinoma. Cancer Biol Med.

[12] Yugawa K, Maeda T, Nagata S, Sakai A, Taketani K, Yamaguchi S (2023). A novel combined carbohydrate antigen 19 – 9 and lymphocyte-to-monocyte ratio score can predict early recurrence of resectable pancreatic ductal adenocarcinoma. Surg Today.

[13] Chen Q, Yin H, Liu S, Shoucair S, Ding N, Ji Y et al. Prognostic value of tumor-associated N1/N2 neutrophil plasticity in patients following radical resection of pancreas ductal adenocarcinoma. J Immunother Cancer. 2022;10(12). 10.1136/jitc-2022-005798 PMC9730407 36600557

[14] Nurmi AM, Mustonen H, Haglund C, Seppanen H (2021). Changes in CRP and CA19-9 during preoperative oncological therapy predict postoperative survival in pancreatic ductal adenocarcinoma. Oncology.

[15] Itoh S, Tsujita E, Fukuzawa K, Sugimachi K, Iguchi T, Ninomiya M (2021). Prognostic significance of preoperative PNI and CA19-9 for pancreatic ductal adenocarcinoma: a multi-institutional retrospective study. Pancreatology.

[16] Murakawa M, Kawahara S, Takahashi D, Kamioka Y, Yamamoto N, Kobayashi S (2023). Risk factors for early recurrence in patients with pancreatic ductal adenocarcinoma who underwent curative resection. World J Surg Oncol.

[17] van Wijk L, de Klein GW, Kanters MA, Patijn GA, Klaase JM (2020). The ultimate preoperative C-reactive protein-to-albumin ratio is a prognostic factor for survival after pancreatic cancer resection. Eur J Med Res.

[18] Inoue D, Ozaka M, Matsuyama M, Yamada I, Takano K, Saiura A (2015). Prognostic value of neutrophil-lymphocyte ratio and level of C-reactive protein in a large cohort of pancreatic cancer patients: a retrospective study in a single institute in Japan. Jpn J Clin Oncol.

[19] De Felice F, Cattaneo CG, Poto GE, Antropoli C, Brillantino A, Carbone L (2024). Mapping the landscape of immunonutrition and cancer research: a comprehensive bibliometric analysis on behalf of NutriOnc Research Group. Int J Surg.

[20] Liang X, Yao S, Lu P, Ma Y, Xu H, Yin Z (2021). The Prognostic Value of New Index (LANR) composed of pre-operative lymphocytes, Albumin, and neutrophils in patients with Resectable Colorectal Cancer. Front Oncol.

[21] Wang S, Wang Y, Zhuang J, Wu Y, Shi W, Wang L (2023). Prognostic significance of index (LANR) composed of preoperative lymphocytes, albumin, and neutrophils in patients with stage IB-IIA cervical cancer. PLoS ONE.

[22] Chun YS, Pawlik TM, Vauthey JN (2018). 8th Edition of the AJCC Cancer staging Manual: pancreas and hepatobiliary cancers. Ann Surg Oncol.

[23] He M, Liu Y, Huang H, Wu J, Wu J, Wang R et al. Serum aspartate aminotransferase is an adverse prognostic indicator for patients with resectable pancreatic ductal adenocarcinoma. Lab Med. 2023. 10.1093/labmed/lmad014 37027310

[24] Jablonska B, Lampe P, Mrowiec S (2020). The influence of nutritional status on the incidence of postoperative complications in patients following distal pancreatectomy. Prz Gastroenterol.

[25] Hu ZI, O’Reilly EM. Therapeutic developments in pancreatic cancer. Nat Rev Gastroenterol Hepatol. 2023. 10.1038/s41575-023-00840-w 37798442

[26] Vezakis I, Vezakis A, Gourtsoyianni S, Koutoulidis V, Polydorou AA, Matsopoulos GK et al. An Automated Prognostic Model for pancreatic ductal adenocarcinoma. Genes (Basel). 2023;14(9). 10.3390/genes14091742 PMC10530933 37761882

[27] Yoon SJ, Park B, Kwon J, Lim CS, Shin YC, Jung W et al. Development of Nomograms for Predicting Prognosis of Pancreatic Cancer after Pancreatectomy: a Multicenter Study. Biomedicines. 2022;10(6). 10.3390/biomedicines10061341 PMC9220008 35740364

[28] Gu Y, Hua Q, Li Z, Zhang X, Lou C, Zhang Y (2023). Diagnostic value of combining preoperative inflammatory markers ratios with CA199 for patients with early-stage pancreatic cancer. BMC Cancer.

[29] Huang JC, Pan B, Jiang T, Zhang XX, Lyu SC, Lang R (2023). Effect of the preoperative prognostic nutritional index on the long-term prognosis in patients with borderline resectable pancreatic cancer after pancreaticoduodenectomy. Front Oncol.

[30] Sadeghi M, Keshavarz-Fathi M, Baracos V, Arends J, Mahmoudi M, Rezaei N (2018). Cancer cachexia: diagnosis, assessment, and treatment. Crit Rev Oncol Hematol.

[31] Gupta D, Lis CG (2010). Pretreatment serum albumin as a predictor of cancer survival: a systematic review of the epidemiological literature. Nutr J.

[32] Danan D, Shonka DC, Selman Y, Chow Z, Smolkin ME, Jameson MJ (2016). Prognostic value of albumin in patients with head and neck cancer. Laryngoscope.

[33] Chen Z, Shao Y, Wang K, Cao W, Xiong Y, Wu R (2016). Prognostic role of pretreatment serum albumin in renal cell carcinoma: a systematic review and meta-analysis. Onco Targets Ther.

[34] Lien YC, Hsieh CC, Wu YC, Hsu HS, Hsu WH, Wang LS (2004). Preoperative serum albumin level is a prognostic indicator for adenocarcinoma of the gastric cardia. J Gastrointest Surg.

[35] Asher V, Lee J, Bali A (2012). Preoperative serum albumin is an independent prognostic predictor of survival in ovarian cancer. Med Oncol.

[36] De Luca R, Gianotti L, Pedrazzoli P, Brunetti O, Rizzo A, Sandini M (2023). Immunonutrition and prehabilitation in pancreatic cancer surgery: a new concept in the era of ERAS® and neoadjuvant treatment. Eur J Surg Oncol.

[37] Liew PX, Kubes P (2019). The Neutrophil’s role during Health and Disease. Physiol Rev.

[38] Zhang CL, Gao MQ, Jiang XC, Pan X, Zhang XY, Li Y (2023). Research progress and value of albumin-related inflammatory markers in the prognosis of non-small cell lung cancer: a review of clinical evidence. Ann Med.

